# Outcomes in patients with Hirschsprung disease following definitive surgery

**DOI:** 10.1186/s13104-018-3751-5

**Published:** 2018-09-04

**Authors:** Gunadi, Stefani Melisa Karina, Andi Dwihantoro

**Affiliations:** Pediatric Surgery Division, Department of Surgery, Faculty of Medicine, Public Health and Nursing, Universitas Gadjah Mada/Dr, Sardjito Hospital, Jl. Kesehatan No. 1, Yogyakarta, 55281 Indonesia

**Keywords:** Constipation, Enterocolitis, Hirschsprung disease, Definitive surgery

## Abstract

**Objective:**

Several pull-through procedures have been described for Hirschsprung disease (HSCR) with varying outcomes. We aimed to describe the outcomes in HSCR patients < 18 year of age who underwent surgical procedures at Dr. Sardjito Hospital, Yogyakarta, Indonesia from January 2013 to December 2014.

**Results:**

We utilized 67 HSCR patients, of whom 49 (73%) were males and 18 (27%) females. Neonatal presentation was seen in 57 cases (85%) and most patients (98.5%) had short-segment HSCR. The clinical manifestations were mainly abdominal distension (94%) and delayed passage of meconium (45%). The most common definitive treatment performed was transanal endorectal pull-through (TEPT) (54%), followed by Soave (18%) and Duhamel (13%) procedures. Enterocolitis occurred in 13% of the HSCR patients after endorectal pull-through, but did not reach a significant level (*p*-value = 0.65), while the constipation rate was significantly higher in HSCR patients who underwent posterior neurectomy compared with those other procedures (OR = 15.5, 95% CI = 1.8–132.5; *p*-value = 0.019). In conclusions, most HSCR patients in Indonesia were diagnosed in the neonatal period and underwent the TEPT procedure. Furthermore, the risk of constipation is increased in HSCR patients following posterior neurectomy compared with other definitive surgical techniques.

## Introduction

Hirschsprung disease (HSCR), which is characterized by the absence of ganglion cells (Meissnerr and Auerbach) along variable lengths of the distal gastrointestinal tract, is a common cause of neonatal intestinal obstruction, which is of great interest to pediatric surgeons throughout the world [1]. This disorder can be classified as follows: (1) short-segment (aganglionosis is confined to the rectosigmoid colon), (2) long-segment (aganglionic segment extends proximal to the sigmoid), and (3) total colonic aganglionosis [1].

Recently, a common variant within the *RET* gene, rs2435357, has been associated with HSCR across populations [2–6]. This variant lies within a conserved transcriptional enhancer of *RET* and has been shown to disrupt a *SOX10* binding site within MCS + 9.7 that reduces *RET* gene expression, an underlying defect in HSCR [2].

The present management for HSCR is removal of the aganglionic segment of the intestines. Several definitive surgeries have been established for HSCR such as transabdominal endorectal pull-through (Soave), Duhamel, transanal endorectal pull-through (TEPT), transanal Swenson-like, and posterior neurectomy procedures [7–11], with varying outcomes [12]. Therefore, we aim to describe the outcomes, constipation and enterocolitis, in HSCR patients following a definitive surgery in Indonesia.

## Main text

### Methods

#### Patients

**Fig. 1:**
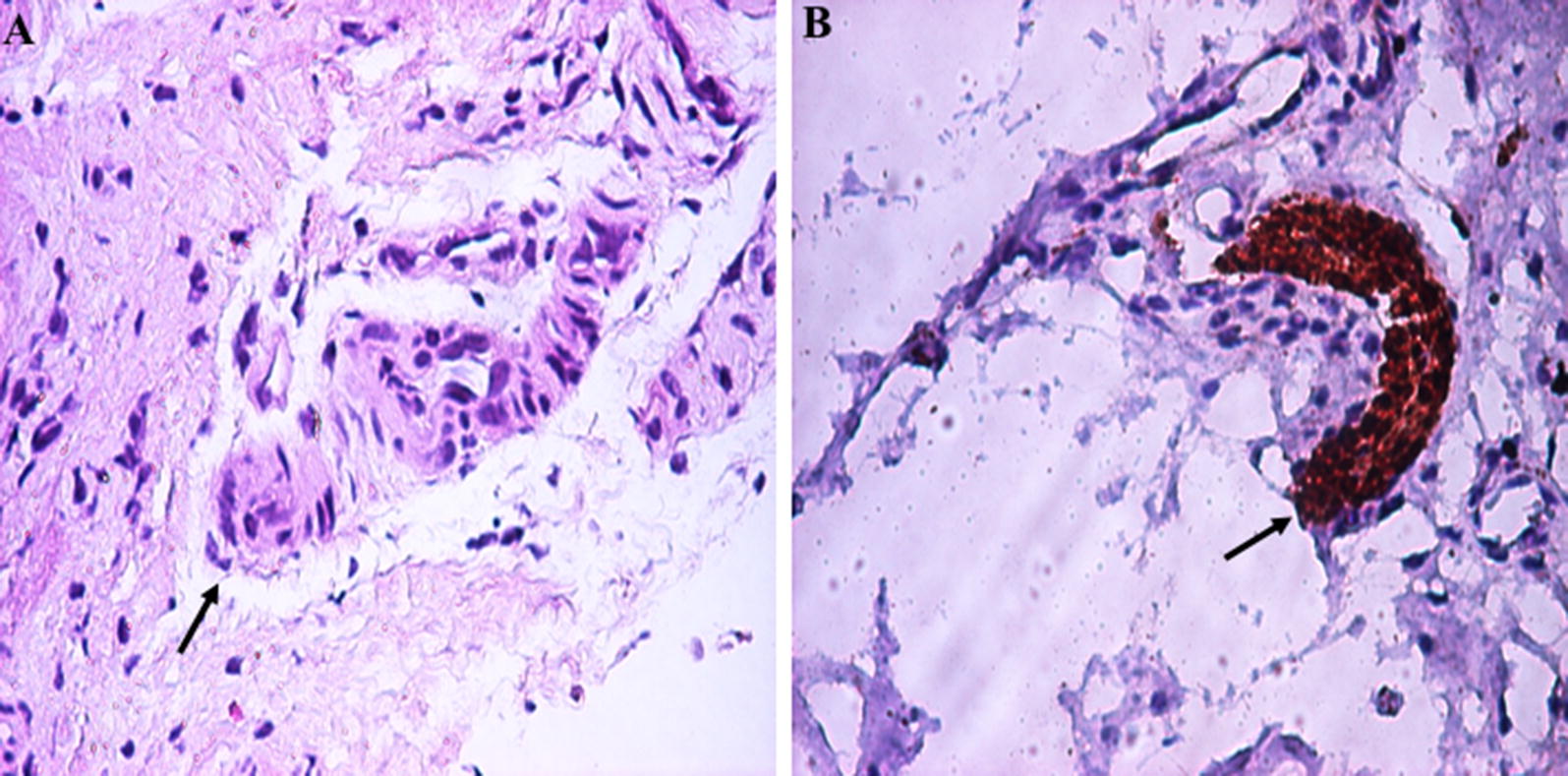
Histopathological findings of full-thickness rectal biopsy in a patient with Hirschsprung disease (HSCR) showed hypertrophic nerve trunk and no ganglion cells (arrow): **a** hematoxylin and eosin staining (×200), **b** S100 immunohistochemistry (×200)

Medical records of histopathologically (Fig. 1) diagnosed HSCR patients in Dr. Sardjito Hospital, Indonesia during the study period of January 2013 and December 2014 were evaluated [13]. For the HSCR incidence calculation, we only utilized the patients from Yogyakarta province.

The study was approved by the Institutional Review Board, Faculty of Medicine, Public Health and Nursing, Universitas Gadjah Mada/Dr. Sardjito Hospital (#KE/FK/232/EC). Written informed consent forms were completed by all parents before participation in this study.

#### Definitive surgical procedures

The Soave, Duhamel, or TEPT techniques were performed at our hospital based on previous studies [7–9], while the posterior neurectomy method has been described in our recent report [11].

#### Constipation and enterocolitis

Constipation was classified according to Krickenbeck category [14], whereas the enterocolitis diagnosis was determined using the Delphi score system [13, 15].

### Results

In this study, we have ascertained 67 HSCR patients of whom 49 and 18 were males and females, respectively. This gives a male-to-female ratio of 2.7:1. The number of new HSCR cases from Yogyakarta province in 2013 was 14, while the number of newborns in 2013 in Yogyakarta province was 45,436 [16]. Therefore, the incidence of HSCR in Yogyakarta, Indonesia based on the annual number of cases divided by the annual number of newborns was approximately 1:3250.

All patients were sporadic HSCR, except one, with degree of aganglionosis as follows: short-segment in 66 (98.5%) patients, long-segment in 1 (1.5%) patients and total colonic aganglionosis was none. The long-segment patient was a sporadic HSCR case and from Yogyakarta province, while the familial HSCR case was also from Yogyakarta province. Neonatal presentation was seen in 56 (84%) patients, whereas 5 (8%), 3 (4%), 2 (3%), and 1 (1%) cases presented as infant, toddler, child, and adolescent, respectively. The clinical manifestations were mainly abdominal distension (94%) and delayed passage of meconium (45%) (Table 1).

**Table 1:** Clinical characteristics of patients with HSCR in Indonesia

Characteristics	N (%)
Gender	
Male	49 (73)
Female	18 (27)
Age distribution	
Neonate	56 (84)
Infant	5 (8)
Toddler	3 (4)
Child	2 (3)
Adolescent	1 (1)
Aganglionosis type	
Short-segment	66 (98.5)
Long-segment	1 (1.5)
Total colonic aganglionosis	0
Clinical manifestation	
Abdominal distension	63 (94)
Delayed meconium passage (> 24 h)	30 (45)

*HSCR* Hirschsprung disease

A definitive surgery was performed in 39 infants, twenty-eight underwent a colostomy and a full-thickness rectal biopsy awaiting pull-through procedure. All cases were histopathologically proven as HSCR prior to any surgery (Fig. 1). The TEPT procedure has been the most common operation (54%), followed by Soave (18%), and Duhamel (13%) procedures. One patient underwent posterior myectomy, while a posterior neurectomy was performed in 13% of the HSCR patients.

Enterocolitis occurred in 15% of the HSCR patients, with the highest rate (13%) after endorectal pull-through, but did not reach a significant level (*p*-value = 0.65) Constipation frequency after surgery was 15%, and most of them (7.5%) underwent posterior neurectomy (*p*-value = 0.019) (Table 2).

**Table 2:** Surgical procedures and outcomes in patients with HSCR in Indonesia

Definitive surgery and outcomes	N (%)	OR (95% CI)	*p*-value
Definitive surgery	39 (58)		
TEPT	21 (54)		
Soave	7 (18)		
Duhamel	5 (13)		
Posterior neurectomy	5 (13)		
Posterior myectomy	1 (2)		
Outcomes after surgery (N = 39)			
Enterocolitis	6 (15)		
TEPT	3 (7.5)	2.2 (0.2–21.1)	0.65
Soave	2 (5)		
Duhamel	1 (2.5)		
Posterior neurectomy	0		
Posterior myectomy	0		
Constipation	6 (15)		
Posterior neurectomy	3 (7.5)	15.5 (1.8–132.5)	0.019*
TEPT	1 (2.5)		
Soave	0		
Duhamel	2 (5)		
Posterior myectomy	0		

Characteristic and surgical outcomes	Yogyakarta province (%)	Outside province (%)	*p*-value
Age distribution	N = 30	N = 37	
Neonate	26 (87)	32 (86)	1.00
Post-neonate	4 (13)	5 (14)	
Outcomes after surgery	N = 17	N = 22	
Enterocolitis	2	4	0.68
Constipation	2	4	0.68

Characteristic and surgical outcomes	Male (%)	Female (%)	*p*-value
Age distribution	N = 49	N = 18	
Neonate	42	16	1.00
Post-neonate	7	2	
Definitive surgery	N = 31	N = 8	
TEPT	16	5	0.70
Soave	7	0	
Duhamel	4	1	
Posterior neurectomy	3	2	
Posterior myectomy	1	0	
Outcomes after surgery	N = 31	N = 8	
Enterocolitis	6	0	0.31
Constipation	3	3	0.09

*HSCR* Hirschsprung disease, *TEPT* transanal endorectal pull-through

*Significant (*p*-value < 0.05)

Furthermore, there were no significant differences between the Yogyakarta and outside province HSCR cases in term of the age distribution of diagnosis, enterocolitis and constipation rates after definitive surgery (*p*-value = 1.00, 0.68, and 0.68, respectively) (Table 2). The age distribution of diagnosis, type of definitive surgery, enterocolitis and constipation rates after surgery were also comparable between male and female HSCR patients (*p*-value = 1.00, 0.70, 0.31, and 0.09, respectively) (Table 2).

### Discussion

We showed evidence that the incidence of HSCR in Yogyakarta, Indonesia is higher than other regions [17, 18], even compared with other Asian countries [19]. It might relate to Indonesian genetic structure ethnicity [20, 21]. Recently, our studies showed that Indonesian controls have a high frequency of *RET* rs2435357 risk allele (0.50) [3], which was higher than those in the European and the African ancestry individuals (0.25 vs. 0.01) [22]. The high incidence of HSCR in Indonesia could also be caused by improved ascertainment due to the establishment of more accurate registry in our hospital or due to advancement in diagnosis and coding over time. However, this data should be interpreted with carefulness since this high incidence may not have a clinical significance [20].

Familial history has been reported in ~ 40% of HSCR cases, particularly with total colon aganglionosis and in female patients [23]. In this study, there was only one patient with family history of HSCR. The patient and affected sibling are females, but presented with short-segment aganglionosis.

The majority of HSCR cases of our study were diagnosed in the neonatal period (85%), which is similar to that of a previous study in Europe (78.5%) [20]. However, our study showed different results from studies from Burkino Faso [17] and Japan [19], where the diagnosis of HSCR was made in the neonatal period in 36% and 40.1–53.4% cases, respectively. Furthermore, in Australia, the percentage of HSCR cases being diagnosed in the neonatal period is higher (90.5%) than in Indonesia [18]. Our study might imply that the HSCR in our hospital has been early diagnosed. Furthermore, our hospital is one of the tertiary referral hospitals in Indonesia. Nevertheless, early diagnosis is important to prevent complications, especially enterocolitis, a significant cause of mortality [24].

Formerly, the neonates diagnosed with HSCR underwent colostomy and waited until 6–12 months later for definitive pull-through. However, this approach has altered greatly over three decades, and the primary pull-through is becoming popular among pediatric surgeons worldwide, in which the TEPT is the most commonly performed procedure [24]. Our study showed a similar trend that the TEPT procedure has been the commonest operation in our hospital.

In this study, the enterocolitis frequency (15%) following definitive surgery was relatively similar with other studies (14–20%) [10, 19], with the highest rate in our HSCR patients (13%) after endorectal pull-through. It has been shown that the enterocolitis is a more complex disease that will not be solely resolved by the type of surgery chosen [13, 24].

The constipation frequency varied from 6 to 34% [25]. Our cohort patients showed post-operative constipation of 15%, which was comparable with a previous study (10%) [12]. In addition, the highest constipation rates were in the patients who underwent posterior neurectomy (7.5%). This result might be due to not performing resection of the aganglionic colon during posterior neurectomy [11].

### Conclusions

Most HSCR patients in Indonesia were diagnosed in the neonatal period and underwent the TEPT procedure. Furthermore, the risk of constipation is increased in HSCR patients following posterior neurectomy compared with other definitive surgical techniques.

## Limitation

A possible limitation of the study involves having only extracted information retrospectively from available medical records.

## Abbreviations

HSCR: Hirschsprung disease
TEPT: transanal endorectal pull-through
